# Seroprevalence of small ruminant caprine arthritis encephalitis lentivirus among goats from selected small ruminant farms in Selangor, Malaysia

**DOI:** 10.14202/vetworld.2018.172-176

**Published:** 2018-02-12

**Authors:** Faez Firdaus Abdullah Jesse, Asinamai Athliamai Bitrus, Yusuf Abba, Veenosha Nehru Raju, Idris Umar Hambali, Innocent Damudu Peter, Abd Wahid Haron, Mohd Azmi Mohd Lila, Jefri Mohd Norsidin

**Affiliations:** 1 Department of Veterinary Clinical Studies, Faculty of Veterinary Medicine, Universiti Putra Malaysia, 43400 Serdang, Selangor, Malaysia; 2 Department of Veterinary Pathology and Microbiology, Faculty of Veterinary Medicine, Universiti Putra Malaysia, 43400 Serdang, Selangor, Malaysia; 3 Institute of Tropical Agriculture and Food security, Universiti Putra Malaysia, 43400 Serdang, Selangor, Malaysia; 4 Department of Veterinary Pathology, Faculty of Veterinary Medicine, University of Maiduguri. P.M.B 1069 Maiduguri, Borno, Nigeria; 5 Department of Veterinary Public Health and Preventive Medicine, Faculty of Veterinary Medicine, University of Maiduguri. P.M.B 1069 Maiduguri, Borno, Nigeria; 6 Department of Theriogenology, Faculty of Veterinary Medicine, University of Maiduguri. P.M. B 1069 Maiduguri, Borno, Nigeria; 7 Research Unit Microbial Food Safety and Antimicrobial Resistance, Department of Veterinary Public Health, Faculty of Veterinary Science, Chulalongkorn University, 10330 Pathumwan, Bangkok, Thailand

**Keywords:** *Caprine arthritis encephalitis*, enzyme-linked immunosorbent assay, goats, lentivirus, seroprevalence, small ruminant farm

## Abstract

**Background and Aim:**

*Caprine arthritis encephalitis* (CAE) is an important viral disease of small ruminants particularly in dairy goats with severe social and economic implication. Hence, this study was designed to determine the seroprevalence of CAE virus (CAEV) among goat population in selected small ruminant farms in Selangor and the risk factors associated with the occurrence of the disease.

**Materials and Methods:**

Blood samples were collected from a total of 91 goats selected at random. Blood serum was harvested and used for competitive enzyme-linked immunosorbent assay test to detect antibodies against CAE virus.

**Results:**

The result obtained showed that 8/91 (8.8%) of the goats were seropositive for CAEV. In addition, biosecurity management, source of origin and sex of the animal were observed to be important risk factors associated with the occurrence of CAE in goats.

**Conclusion:**

The findings of this study affirmed that the seroprevalence of CAEV infection among goat population in small ruminant farms in Selangor, Malaysia, is low. However, there is need to institute strict control measures such as testing and culling positive animals or separation of infected animals from those that tested negative to the disease for effective eradication of the disease.

## Introduction

*Caprine arthritis encephalitis* (CAE) is a severe and chronic devastating disease of goats caused by a lentivirus that is characterized by significant economic loss [1,2]. This may have led to a negative impact on milk production and increasing the risk of mastitis development [3,4]. CAE virus (CAEV) infection is manifested as polyarthritis in adult goats and encephalitis in kids [4]. In addition, the disease is one of the major problems of modern farming facilities with intensive production [5]. CAEV has a worldwide distribution and is commonly prevalent among goat population irrespective of all the measures instituted against it [6]. The disease is caused by a Lentivirus of the Retroviridae family. The disease is mainly characterized by encephalitis and painful arthritis in goats of all ages, gender, and breed. However, other clinical signs of the disease include swelling of the joint capsule which subsequently leads to lameness. Signs of mastitis, synovitis, reduced growth rate, and pneumonia have also been reported in cases of CAE virus infection [6,7]. The virus has the ability to infect both dividing and none dividing cells, a feat that makes it an ideal viral vector for neuroscience [7]. The virus is transmitted both vertically by ingestion of milk and horizontally by direct contact with infected animals [4,6,7]. CAEV can also be transmitted to sheep in farms where goats and sheep are raised together; hence, indicating interspecies transmission [8]. Small ruminant lentivirus (SRLV) can be divided into six sequence Clades (I-VI) as defined by early phylogenetic studies. The ­Icelan­dic visna virus and other closely related members of the Maedi-visna virus strains belong to Clade I, while the North American lentivirus isolated from sheep belong to Clade II. Clades III and IV contain the French and Norwegian small ruminants lentivirus, while Clade V contains both Swiss and French CAEV strains. Finally, the French SRLV belongs to Clade VI. Recently, a more accurate classification based on the longer genetic sequences has divided the CAEV into four major sequences of Groups A-D. The Group A contains about seven subtypes while Group B contains only two subtypes. To date, subtypes A5 and A7, and Groups C and D have been found only in goats. In addition, other subtypes such as A1 and A2 have been isolated only from sheep. Furthermore, subtypes A3, A4, A6, B1, and B2 have been isolated from sheep and goat [3,9].

The diagnosis of the disease involves a combination of clinical manifestation, postmortem examination, and histopathological findings. However, serology is considered as one of the easiest and most efficient ways to diagnose infections caused by CAEV [7]. In practice, the most common serological test adopted for the diagnosis of CAEV infection is enzyme-linked immunosorbent assay (ELISA) and agar gel immunodiffusion (AGID) [9]. However, ELISA test is the most preferred due to the low sensitivity associated with AGID [7]. Studies have also shown that CAEV is widely spread among dairy goat especially in the developed countries such as United States, Canada, France, Norway, and Switzerland, where the seroprevalence of more than 61% was reported by Ling *et al*. [10]. In addition, several authors have reported the global distribution of CAEV, for instance, Adams *et al*. [11] reported that the prevalence rate of CAEV in Kenya and America was 4.5% and 81%, respectively. Similarly, Lilenbaum *et al*. [12] and Bandeira *et a*l. [3] reported that the prevalence of CAEV in Brazil ranges from 8.2% to 14.1%, while Ghanem *et al*. [9] reported a 6.0% prevalence rate in Northern Somalia. In the Middle East, even though there is paucity of data on the seroprevalence of CAEV especially in the Gulf States; Al-Qudah *et a*l. [13] reported that 12.2% of goats in Jordon are seropositive to CAEV, while only 0.8% of sheep in Saudi Arabia were reported to be seropositive to CAEV [14].

In Malaysia, the disease was first reported in 2010 by Noordin *et al*. [15], where 20 goats from a herd of 2000 in a farm manifested nervous signs characteristics of CAE. Four goats were brought to the University Veterinary Hospital, Universiti Putra Malaysia (UPM). The disease was diagnosed using clinical signs, pathology and isolation of CAEV. Similarly, a few years later, Ling *et al*. [10] also reported a case of suspected CAE in a Boer cross kid which was diagnosed according to clinical signs, postmortem, and histopathological findings. Since then, to the best of our knowledge, there was no report of CAEV infection in Malaysia.

This study was designed to determine the seroprevalence of CAE among goat population in selected small ruminant farms in Selangor, Malaysia and to evaluate the risk factors that are associated with the occurrence of the disease.

## Materials and Methods

### Ethical approval

This study was performed according to the guidelines for the care and use of animals as approved by Institutional Animal Care and Use Committee of UPM, Animal Welfare Act (2014) [AUP No.: FYP.2016/FPV (32.50)].

### Samples collection

A total of 91 goats were enrolled in this study from five small ruminant farms in Selangor, Malaysia. The goats were sampled based on age (1<, 1-6 years and >6-year-old), sex (males n=5 and females n=86), history of biosecurity management in the farms, source of the animals (exotic or local), and breeds. A total of n=56 of the goats in each farm were between the ages of 1 and 6 years, n=66 of the animals were imported flocks. In addition, n=41 of all the goats in each sample were Saanen breed. Blood (10 mL) was obtained from each goat through jugular venipuncture into pre-labeled vacutainer tubes (5 mL each into plain and sodium heparin tubes to harvest serum and plasma, respectively) and transported in an ice box to the laboratory for serology.

### Serum sample preparation

Blood samples were centrifuged at 3000× g for 5 min, after which the harvested serum sample was transferred into a 1.5 mL microcentrifuge tube (Eppendorf). Then, the serum samples were stored at -20°C temperatures until when used.

### Competitive inhibition ELISA (cELISA) test

All serum samples were screened for anti-CAEV antibodies using a commercially available cELISA (VMRD Inc., Pullman, WA, USA). Each test kit included positive and negative goat sera verified by immunoprecipitation. The principle of this protocol was as described by Herrmann *et a*l. [16]. Briefly, the competitive assay was conducted based on the displacement of horseradish peroxidase-conjugated MAb GPB 74A by antibodies present in the undiluted test goat sera. Results were expressed as percent inhibition of MAb GPB74A binding, whereby percent inhibition=100−(sample O.D.×100)/(mean negative control O.D.). Samples producing ≥35% inhibition were considered positive. The test has been reported to have a 100% sensitivity and 99.6% specificity [7,16,17].

### Risk factors

The factors investigated to assess their association with the seroprevalence of CAEV in goats are age, sex, breeds, biosecurity management, and the sources of the animals. The results were considered statistically significant at p≤0.05 at a 95% confidence interval.

### Statistical analysis

The seroprevalence of CAEV in goats was determined using the IBM SPSS Statistics Version 22 Software. Pearson Chi-square was used to determine the association between risk factors and the seroprevalence of CAEV among goat population in this study. PHI and Cramer’s V test was used to determine the strength of association between the risk factors and seropositivity to CAEV.

## Results

**Figure-1:**
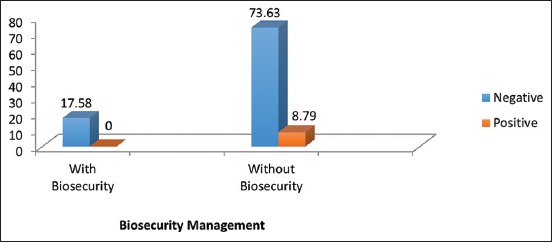
Seroprevalence of *Caprine arthritis encephalitis* among goats in selected farms based on biosecurity management.

The result obtained in this study showed that the seroprevalence rate of CAEV among goats was 8.8% (8/91). Among the 5 farms sampled 4 farms had animals that were seropositive for CAEV (Table-1 and Figure-1). The calculation of the true prevalence as described by Toma *et al*.[18], was 8.42%.

**Table-1:** Seroprevalence of the CAEV among goats from selected farms in Peninsular Malaysia based on breeds, source, age, and sex.

Variables	No. tested	No. positive	Prevalence (%)
Farms			
A	25	2	8.0
B	26	0	0.0
C	10	3	30.0
D	21	2	9.5
E	9	1	11.1
Breeds			
Saanen	41	4	4.4
Saanen cross	6	0	0.0
Jamnapari	5	0	0.0
Jamnapari cross	15	0	0.0
Boer cross	21	4	4.4
Gurum	3	0	0.0
Source			
Imported	66	8	8.8
Local	13	0	0.0
Unknown	12	0	0.0
Age			
<1	24	2	2.2
1-6	56	6	6.6
>6	11	0	0.0
Sex			
Male	5	2	2.2
Females	86	6	6.6

CAEV=*Caprine arthritis encephalitis virus*

### Risk factor analysis

Analysis of risk factors using Chi-square at p<0.05 showed that there was a statistically significant association between sex of the animal and the seroprevalence of CAEV among goat populations where 6.6% (6/91) of the female goats were seropositive for CAEV compared to the 2.2% (2/91) in male goats (Table-1). In addition, no statistically significant association existed between the breed of goats, biosecurity management, age, and source of an animal with the seroprevalence of CAEV p > 0.05. However, seropositivity against CAEV was observed more in animals between the ages of 1 and 6 years, imported animals and in farms where biosecurity management is not practiced (Table-1 and Figure-1). Similarly, 4.4% (4/91) which constituted about 50% each of the total number of goats that were seropositive for CAEV belong to Saanen and Boer crossbreeds of goats, whereas other breeds sampled were seronegative against CAEV. However, there is no statistically significant association (p>0.05) between breed of the animals and the seroprevalence of CAEV (Table-1).

## Discussion

CAE is a chronic debilitating and economically important viral disease of goats. This is because the virus infects its host for life. In most cases, the disease condition manifested as subclinical; however, a small number of the animals develop progressive, untreatable disease that includes encephalitis in kids and polyarthritis in adult goats [6]. In addition, the disease decreases lifetime productivity in dairy goats, particularly when the prevalence of infection is high within a herd [17]. In addition, CAE has been reported as a barrier to the exportation of goats from countries where it is endemic, including countries like the United States [17]. This study revealed that the herd seroprevalence of CAEV among goat population in selected small ruminant farms in Selangor, Malaysia, was 8.42%. This was considered to be 9 times lower than the prevalence of CAEV reported in the United States [6]. The disparity, however, can be attributed to epidemiological factors such as difference in climate, the number of animals sampled and the sensitivity and specificity of the test method used. In addition, studies have also shown that CAEV has worldwide distribution and is mostly seen in developed countries where intensive goat management is practiced [6]. This was further supported by the findings of OIE, [19] where it was reported that countries such as Canada, Australia, New Zealand, France, Sweden, and China have a prevalence of more than 65%. Seroprevalence rate of 8.42% obtained in this study is still low compared to other countries, yet this value only represents the 5-selected small ruminant farms in the Selangor state, Malaysia. Serological testing against CAEV needs to be done in each state of Malaysia to know the true seroprevalence status of CAEV in the country. In addition, detection of the virus using molecular techniques and large sample population will also help to determine the true prevalence of the virus.

The higher prevalence rate of CAEV reported in few countries are associated with risk factors such as herd management, breed of goats, size of herd, and age of the animal [20]. This agrees with the outcome of this study where seropositivity to CAEV is observed more in exotic breeds 8.8% (8/91), goats between the ages of 1-6 years 6.6% (6/91) and in farms where biosecurity measures are not practiced (Table-1 and Figure-1). Similarly, in this study, a significant association between sex and seroprevalence of CAEV was observed. A much higher number of does 6.6% (6/91) were seropositive against CAEV than the bucks 2.2% (2/91). This finding is, however, not in agreement with the work of Grewel *et al*. [21] Where the authors reported a higher seroprevalence in bucks than in doe. The difference could be due to the number of goats sampled as well as the sex of the animals in each sampled population. Similarly, Bandeira *et al*. [3] reported that regardless of age, bucks (28.3%) were found to be more frequently seropositive for CAEV than does (5.9%). Higher seropositivity was seen in Saanen and Boer cross goats in this study, which may be due to the higher sample collected from these two breeds. However, there is no significant association between the breed of goat and seroprevalence toward CAEV. The result from this study is not in agreement with the work of Greenwood *et al*. [22] where lower seroprevalence of CAEV was reported in Saanen breed in a study done in South Wales. Adams *et al*. [23] also reported lower seroprevalence in Golden Guernsey goats than other breeds, and no evidence of infection in Toggenburg goats, but apparently not all breeds had equal opportunity for exposure. Other cross-sectional studies have failed to demonstrate a consistent association of breed with CAEV infection [24].

Farms that do not practice biosecurity management showed higher seropositive result compared to farms that practice biosecurity management in this study. This supports the work by Al-Qudah *et a*l. [13] where the authors reported that poor biosecurity and sanitary management practice is more likely to facilitate the transmission of CAEV infection. The farm that practiced biosecurity management has a very limited access to any outside turnout. In this study, all the seropositive goats were imported from overseas; however, their original source could not be determined due to limited information obtained from farm owners during sample collection. Imported goats can be carriers and spread the disease to the herd before it can be detected. Thus, it is recommended for farms to ensure the practice a strict quarantine protocol before importation of goats so as to avoid introduction of the disease into the herd.

For the age factor, seropositivity to CAEV was seen more in animals between the ages of 1-6 years old compared to another age group in this study. In a study conducted by Al-Qudah *et a*l. [13], it was reported that goats older than 3 years were more likely to be CAEV seropositive than younger goats. However, CAEV has been reported to affect goats of all ages, breeds, and sex [7]. High rate age-related seroprevalence of CAEV has been reported in few studies. This could occur as a result of lifelong infection plus continued transmission, resulting in increased cumulative proportion of infected animal with age [24].

## Conclusion

Herd seroprevalence of CAEV among goat population in selected small ruminant farms from Selangor, Malaysia, is 8.42%. Risk factors associated with seroprevalence at herd level were sex of the animals and farms that do not practice biosecurity management. From this study, it is recommended to have serological testing against CAEV infection in each state of Malaysia to know the true seroprevalence status of CAEV in the country. In addition, genotyping of CAEV should be employed using larger sample size.

## Authors’ Contributions

FFAJ design the research study, AAB drafted the manuscript, YA analyzed the data and revised the manuscript, VNR collect data for the study, IUH and IDP reviewed the manuscript, AWH and MAML reviewed and approved the final draft of the manuscript, while JMN provided technical assistance. All authors read and approved the final manuscript.
